# Higher healthcare use before paediatric multiple sclerosis onset: a nationwide cohort study

**DOI:** 10.1093/braincomms/fcaf180

**Published:** 2025-05-07

**Authors:** Kyla Anne McKay, Ali Manouchehrinia, Feng Zhu, Yinshan Zhao, Ruth Ann Marrie, Colleen Jean Maxwell, Jan Hillert, Helen Tremlett

**Affiliations:** Department of Clinical Neuroscience, Karolinska Institutet, Stockholm 171 77, Sweden; Centre for Molecular Medicine, Karolinska Hospital, Stockholm 171 76, Sweden; Department of Clinical Neuroscience, Karolinska Institutet, Stockholm 171 77, Sweden; Alberta Health Services, Calgary, Canada T5J 3E4; Faculty of Medicine (Neurology), University of British Columbia, Vancouver, Canada V6T 2B5; Faculty of Medicine (Neurology), University of British Columbia, Vancouver, Canada V6T 2B5; Department of Medicine, Dalhousie University, Halifax, Canada B3H 4R2; School of Pharmacy, Faculty of Science, University of Waterloo, Waterloo, Canada N2G 1C5; Department of Clinical Neuroscience, Karolinska Institutet, Stockholm 171 77, Sweden; Faculty of Medicine (Neurology), University of British Columbia, Vancouver, Canada V6T 2B5

**Keywords:** multiple sclerosis, paediatric, prodrome

## Abstract

Evidence of increased healthcare use occurring before paediatric-onset multiple sclerosis presentation suggests a prodromal phase. However, little is known of its duration or features, and few studies have accessed a clinical cohort to examine the period before symptom onset. We compared annual rates of healthcare use before paediatric multiple sclerosis onset in clinical and administrative cohorts versus matched non-multiple sclerosis cohorts. We identified persons with paediatric-onset multiple sclerosis from the Swedish Multiple Sclerosis registry and population-based administrative data using a validated algorithm requiring ≥3 hospital or outpatient multiple sclerosis diagnostic codes recorded on separate dates. The index date was multiple sclerosis symptom onset, as recorded in the multiple sclerosis registry by a neurologist (clinical cohort) or the earliest demyelinating disease-related International Classification of Diseases code (administrative cohort). Individuals with age at index <18 years were matched with up to five individuals from the general population on sex, birth year, county of residence at the index date and residency time. Healthcare use was measured as hospital/outpatient diagnoses (International Classification of Diseases chapters) and prescription drug classes (Anatomical Therapeutic Chemical classification system, 2nd level). Yearly rates of hospital and outpatient visits (up to 17 years pre-index) and prescription fills (up to 14 years pre-index) were compared using Quasi-Poisson regression. The clinical/administrative cohorts included 233/206 paediatric-onset multiple sclerosis and 1151/1011 matched individuals, with a mean age at the index of 16 years (standard deviation: 2) in all four groups. In both cohorts, individuals with paediatric-onset multiple sclerosis exhibited elevated healthcare use predominantly 1–10 years pre-index, including, for example, higher prescriptions filled for corticosteroids for dermatological use (rate ratio range: 2.61–3.91) and outpatient visits for ill-defined signs/symptoms (rate ratio range: 2.17–8.64) and unassigned ICD codes (rate ratio range: 2.20–4.17). In the year pre-index, individuals with paediatric-onset multiple sclerosis in both cohorts exhibited higher rates of outpatient visits for neoplasms, nervous system disorders, sense organ conditions, ill-defined signs/symptoms and ‘other health system contact’ (rate ratio range: 2.05–18.00). In the same year, the clinical cohort also had higher rates of prescription fills for ‘other gynecologicals’ (4.08, 95% confidence interval: 1.04–16.09), and the administrative cohort had higher rates for prescriptions filled across eight drug classes (rate ratio range: 1.56–6.49). Healthcare use was higher primarily in the 1–10 years before paediatric-onset multiple sclerosis versus a matched cohort, suggestive of a prodromal phase. During this period, the paediatric-onset multiple sclerosis cohort was more often identified as having ill-defined signs/symptoms, neoplasms and skin-related issues.

## Teaser

McKay *et al.* demonstrated that individuals with paediatric-onset multiple sclerosis exhibited increased healthcare use 1–10 years before symptom onset. Elevated rates included outpatient visits for ill-defined symptoms, neoplasms and skin-related issues, alongside higher prescription fills. Findings highlight early warning signs, aiding in earlier recognition of paediatric-onset multiple sclerosis and future preventive efforts.

## Graphical Abstract

**Graphical Abstract:**
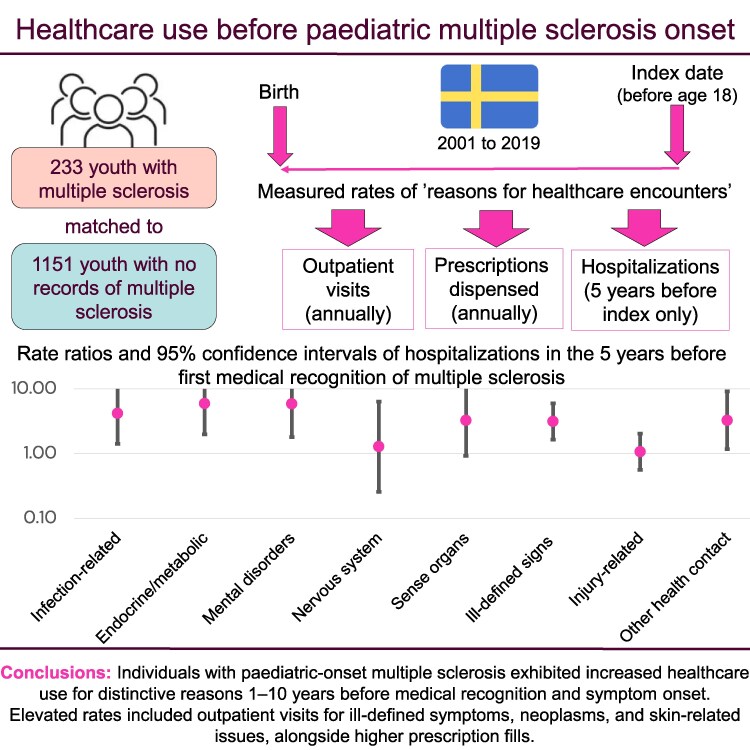

## Introduction

Multiple sclerosis (MS) is a debilitating neurological condition that typically begins in early adulthood, but approximately 5% of patients experience their first symptom in childhood. Awareness and recognition of paediatric-onset MS (PoMS) remain a challenge,^1^ and knowledge surrounding the earliest signs of the disease is suboptimal.^2^ In the general MS population, mounting evidence has suggested the presence of a prodromal phase, measurable in the increased use of health services in the 5–10 years before clinical recognition of the disease.^3^ Elevations in serum neurofilament light levels have also been reported during this period, suggestive of underlying neuroaxonal damage.^4,5^

PoMS may represent the ideal population in which to assess the earliest signs of MS (the prodrome). With fewer years lived, a child has fewer ‘irrelevant’ life events, less exposure to negative health behaviours and fewer comorbidities relative to adults. Recently, a study from Germany reported elevations in primary care visits before the first medical recognition of paediatric MS.^6^ Nine unique disorders, signs and symptoms were more frequently diagnosed in the PoMS population in the 5 years before the first CNS-related demyelinating event, determined using administrative data. Yet much remains unknown, including how long these elevations in healthcare use extend back in time beyond the 5 years examined, whether they encompass additional forms of healthcare contact like secondary care and medication use, and if these patterns are also measurable before MS symptom onset as determined clinically by a neurologist.

In this study, we aimed to identify and characterize health services use, including secondary care and prescription medication information, before the clinically determined MS symptom onset date or first CNS demyelinating event in the PoMS population in Sweden.

## Materials and methods

### Data sources

Nationwide, population-based health administrative data, including hospital and outpatient physician visits and prescriptions dispensed from community pharmacies, were available from the National Patient Register^7^ and Prescribed Drug Registry,^8^ respectively. Demographic information, including sex, birth dates, immigration/emigration dates, vital status and county of residence, were drawn from the Total Population Register.^9^ Annualized family income was obtained from the Longitudinal Integrated Database for Health Insurance and Labour Market Studies.^10^ The Swedish MS Registry, a national quality register that captures an estimated 85% of Sweden's prevalent MS population, provided MS symptom onset date, as recorded by a neurologist.^11^ Use of the registry is voluntary for both neurologists and patients. Data from all sources except prescription drug data were available from 1 January 2001, until 31 December 2019; prescription drug data were available from I July 2005.

### Study population

Two cohorts were created using different definitions of paediatric-onset MS. A clinical cohort included all persons registered in the Swedish MS Registry with an MS symptom onset date recorded by the neurologist before the age of 18 years. The index date for the clinical cohort corresponded to the earliest MS symptom onset date or the first dated CNS demyelinating International Classification of Diseases (ICD) code (ICD-9: 340, 377D, 323X, 341A, 341W, 341X or ICD-10: G35, H46-H48, G37.3, G36), whichever came first. An administrative cohort was established using a validated algorithm [positive predictive value (PPV) of 98.8%) applied to linked health administrative data, which required ≥3 outpatient or hospital-related MS diagnosis codes (ICD-9/10-SE 340/G35) recorded on separate days.^12^ The index date for the administrative cohort was defined as the first CNS-related demyelinating disease ICD code, which must have occurred before the age of 18 years. An individual could fulfil both definitions and appear in both cohorts, but cohorts were analysed separately. A person with MS symptom onset before age 18 (as recorded in the Swedish MS Registry) whose MS was not medically recognized (i.e. had no CNS demyelinating codes recorded) until age 18 or later would be included only in the clinical cohort, not the administrative cohort.

To ensure the inclusion of new-onset PoMS, all individuals in the study must have been residents of Sweden for at least 5 years before their index date. Additionally, no recorded diagnoses of CNS demyelinating diseases prior to the index date were allowed, confirming that cases were incident rather than pre-existing. Each individual with MS was matched with up to 5 individuals from the general population on sex, birth year, county of residence at the index year and residency time in Sweden before the index date. The matched cohort also had to be resident in Sweden until their corresponding person with MS met the validated algorithm for MS (administrative cohort, third MS code) or was diagnosed and registered with the Swedish MS registry (clinical cohort, diagnosis date).

### Outcomes

Rates for healthcare use were compared between the MS and matched cohorts, assessed as hospital or physician outpatient visits, by ICD-10 chapters (based on the primary code reported on the discharge or billing report, see Supplementary Table 1) and prescriptions dispensed, grouped according to the World Health Organization's Anatomical Therapeutic Chemical (ATC) classification system, 2nd level (therapeutic drug class, see Supplementary Table 2). Small numbers of events precluded us from exploring specific diagnoses/drugs within each chapter/drug class. Hospital visits were measured in the 5-year pre-index date period, then annually as far back pre-index as was feasible. Outpatient visits and prescriptions dispensed were measured annually pre-index back to birth, if possible.

### Statistical analysis

Annual rates (or 5-year period for hospitalizations) for each outcome were compared between the MS and matched cohorts using Quasi-Poisson regression. To account for variable residency time each year, the log of residency time was included as an offset in the model. Findings were reported as rate ratios (RRs) and 95% confidence intervals (CIs). We only presented periods with at least 10 events (total among the MS and matched cohorts). Analyses were conducted using R (v4.0.5, R Foundation for Statistical Computing, Vienna, Austria).

### Ethics

Ethical approval was provided by the Swedish Ethical Review Authority and the University of British Columbia's Clinical Research Ethics Board.

## Results

The clinical cohort included 233 individuals with PoMS and 1151 matched individuals; the administrative cohort included 206 and 1011, Table 1. The majority of the PoMS patients were included in both groups (*n* = 178). Fifty-five were identified only in the clinical cohort and 28 only in the administrative cohort. Females predominated (representing 70.0% in the clinical cohort and 65.0% in the administrative cohort). The mean age at the index date in both cohorts was 16 (SD: 2) years. Nine per cent of individuals in the clinical cohort (21/233) had a CNS demyelinating ICD code assigned as the index date as it preceded their MS symptom onset date (*n* = 14), or their MS symptom onset date was not recorded (*n* = 7). Among those in both cohorts, the median (IQR) time difference between the clinical and administrative index dates was 76 (12–203) days. For the analysis of prescriptions dispensed, the cohorts were smaller due to the later start of the drug registry and comprised 140 PoMS and 686 matched individuals in the clinical cohort and 141/686 in the administrative cohort. Demographic and clinical characteristics for the 4 (non-mutually exclusive) groups are available in Table 1 and Supplementary Table 3.

**Table 1:** Demographic and clinical characteristics of the administrative and clinical paediatric-onset multiple sclerosis (MS) and matched cohort

	Clinical cohort		Administrative cohort	
	MS	Matched cohort	MS	Matched cohort
N	233	1151	206	1011
**Sex, *N* (%)**				
Female	163 (70.0)	801 (69.6)	134 (65.0)	656 (64.9)
**Age at index**				
Mean (SD)	15.9 (1.9)	16.0 (1.8)	16.1 (1.8)	16.2 (1.8)
**Age at diagnosis**				
Mean (SD)	17.6 (2.9)	17.7 (2.9)	17.0 (2.5)	17.1 (2.5)
**Age at index (categories), *N* (%)**				
<12	10 (4.3%)	47 (4.1%)	9 (4.4)	33 (3.3%)
12–15	43 (18.5%)	408 (35.4%)	70 (34.0)	188 (18.6%)
16–<18	180 (77.3%)	696 (60.5%)	127 (61.7)	790 (78.1%)
**Index year (categories), N (%)**				
2001–2010	100 (42.9)	500 (43.4%)	74 (35.9)	370 (36.6)
2011–2020	133 (57.1)	651 (56.6%)	132 (64.1)	641 (63.4)
**Socioeconomic status** ^a^ **, N (%)**				
1 (lowest income quintile, least affluent)	23 (9.9)	66 (5.7%)	17 (8.3)	72 (7.1)
2	28 (12.0)	95 (8.3%)	25 (12.1)	64 (6.3)
3	38 (16.3)	169 (14.7%)	41 (19.9)	145 (14.3)
4	52 (22.3)	310 (26.9%)	44 (21.4)	265 (26.2)
5 (highest income quintile, most affluent)	92 (39.5)	511 (44.4%)	79 (38.3)	465 (46.0)
**Immigrant status**				
Immigrant	13 (5.6%)	36 (3.1%)	15 (7.3%)	36 (3.6%)
Non-immigrant	220 (94.4%)	1115 (96.9%)	191 (92.7%)	975 (96.4%)
**Follow-up before index, years**				
Mean (SD)	10.3 (3.3)	10.5 (3.4)	10.8 (3.4)	11.1 (3.4)

PoMS, paediatric-onset multiple sclerosis; SD, standard deviation;.

^a^The socioeconomic status of the highest-earning parent was used. 11 controls in the administrative cohort and 14 controls in the clinical cohort with missing parental socioeconomic status were assigned to category 3.

### Clinical cohort

#### Hospital visits

**Figure 1:**
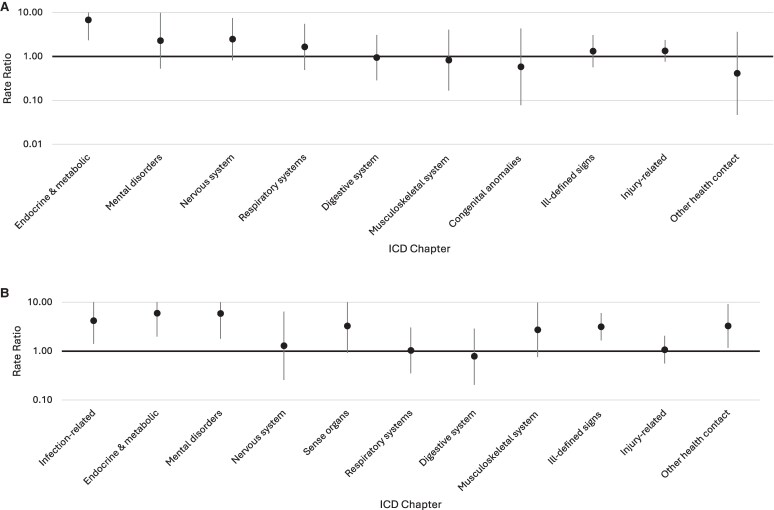
**Rate ratios (RRs) and 95% confidence intervals of ICD-chapter-specific hospitalizations in the 5-year period before MS symptom onset (A) and first demyelinating claim (B)**. RR >1 indicates higher number of hospitalizations among the PoMS versus the matched cohort. Chapters with too few events to enable model convergence are not included. Upper 95% confidence intervals extend beyond the figure in some chapters.

In the 5-year pre-MS symptom onset, hospitalizations were elevated in the PoMS clinical cohort for endocrine and metabolic-related reasons only (6.79, 95% CI: 2.35–19.64, Fig. 1A). Low number of events prevented yearly analyses of hospital visits.

#### Outpatient physician visits

Yearly RRs for outpatient visits were significantly elevated for specific ICD chapters before clinical onset (Supplementary Fig. 1). Not all ICD chapters could be explored in every year due to small numbers of events. In the year before onset, outpatient visits relating to neoplasms, the nervous system, sense organs, ill-defined signs and symptoms and ‘other health system contact’ were higher in the PoMS group (RRs ranged from 2.78 to 4.23). In years 8 to 10, visits for which no ICD code was assigned were elevated in the PoMS group (RRs ranged from 2.39 to 3.09). Eight and 14 years before the index date, the PoMS group exhibited higher rates of injury-related outpatient visits (RRs ranged from 2.69 to 3.21). While 16 years before the index date, the PoMS cohort exhibited higher rates of infections (5.42, 95% CI: 1.61–18.29), though this was based on just 5 events in the PoMS group (Table 2). Neoplasms were elevated every year for 6 years before clinical onset, reaching statistical significance in the 1 and 4 years pre-index (RRs: 2.96 and 4.18, respectively). While skin-related visit RRs were consistently elevated in each of the 4 years pre-index date, none met statistical significance.

**Table 2:** Annual rate ratios of outpatient visits by ICD chapters in the clinical cohorts (paediatric-onset MS versus matched cohort), only significant differences (at *P* < 0.05) reported

Year before index	ICD chapter	Chapter description	Events (PoMS cohort)	Events (matched cohort)	Rate ratio (95% CI)
1	2	Neoplasms	9	15	2.96 (1.04–8.47)
1	6	Nervous system	18	32	2.78 (1.08–7.15)
1	7	Sense organs	36	42	4.23 (2.00–8.95)
1	17	Ill-defined signs and symptoms	48	73	3.25 (2.04–5.17)
1	19	Other health system contact	59	98	2.97 (1.89–4.69)
4	2	Neoplasms	11	13	4.18 (1.40–12.49)
8	18	Injury-related	26	49	2.69 (1.43–5.07)
8	None	ICD code unassigned	32	68	2.39 (1.07–5.31)
9	None	ICD code unassigned	44	79	2.84 (1.26–6.42)
10	None	ICD code unassigned	33	55	3.09 (1.35–7.07)
14	18	Injury-related	<5	7	3.21 (1.03–9.99)
16	1	Infection-related	5	5	5.42 (1.61–18.29)

Cell sizes <5 are suppressed as per data access and privacy requirements.

#### Prescription drug classes dispensed

Up to 9 years before MS symptom onset, there were differences in prescription RRs between the PoMS and matched cohort, although event rates each year were modest, ranging from 5 to 8 for the PoMS cohort. Drug classes, which were significantly higher for the PoMS cohort included other gynecologicals (including contraceptives for topical use) in the year before the index date (4.08, 95% CI: 1.04–16.09), sex hormones and modulators of the genital system 4 years pre-index date (3.27, 95% CI: 1.16–9.20) and corticosteroids for dermatological use 6 and 9 years pre-index date, RRs 3.46 and 3.18, respectively (Table 3).

**Table 3:** Annual rate ratios of prescription drug classes dispensed (at ATC 2nd level) in the clinical cohort (paediatric-onset MS versus matched cohort), only significant differences (at *P* < 0.05) reported

Year before index	ATC code	Prescription drug class (ATC 2nd level)	Events (PoMS cohort)	Events (matched cohort)	Rate ratio (95% CI)
1	G02	Other gynecologicals	5	6	4.08 (1.04–16.09)
4	G03	Sex hormones and modulators of the genital system	8	12	3.27 (1.16–9.20)
6	D07	Corticosteroids (dermatological)	7	10	3.46 (1.17–10.28)
9	D07	Corticosteroids (dermatological)	5	8	3.18 (1.05–9.61)

### Administrative cohort

#### Hospital visits

In the 5-year pre-index period, hospital visits were higher in the PoMS cohort for reasons relating to infections, the endocrine and metabolic system, mental disorders, ill-defined signs and symptoms and ‘other health system contact’ which includes visits for which no diagnosis had been made, but further investigations were being conducted (RRs ranged from 3.16 to 5.96, Fig. 1B). The analyses of yearly RRs for hospital visits were limited to the first few pre-index years due to the small number of events. Only endocrine and metabolic-related disorders (RR: 11.45, 95% CI: 1.92–68.25) and ill-defined signs and symptoms (3.68, 95% CI: 1.39–9.72) were elevated in the year pre-index. There were no other significant differences between groups in yearly hospitalization rates.

#### Outpatient physician visits

Outpatient visits for specific ICD chapters showed differences between PoMS and matched individuals in multiple chapters (Supplementary Fig. 2).

In the year before onset, outpatient visits relating to several ICD chapters, including infections, neoplasms, endocrine and metabolic-related disorders, nervous system, sense organs, ill-defined signs and symptoms, ‘other health system contact’ and visits for which the physician could not, or did not, assign an ICD code, were all elevated (RRs ranged from 2.20–18.00). Among the highest were those related to sense organs (18.00, 95% CI: 10.31–31.42) and nervous system (8.94, 95% CI: 4.20–19.02).

Between 2 and 10 years before the index date, the PoMS cohort experienced significantly higher rates of outpatient visits for neoplasms, the genitourinary system, skin-related issues, ill-defined signs and symptoms, ‘other health system contact’ and visits for which no ICD code was assigned (RRs ranged from 2.04 to 5.19). At 16 years before the index, infection-related outpatient visits were higher among PoMS, although this was based on a limited number of events (7) in the PoMS group (4.33, 95% CI: 1.54–12.18). RRs for diseases of the genitourinary system were consistently higher in the PoMS group, but only met statistical significance 6 years pre-index (Supplementary Fig. 2).

Rate ratios and confidence intervals of significant findings (at *P* < 0.05 level) by ICD chapters are available in Table 4.

**Table 4:** Annual rate ratios of outpatient visits by ICD chapters in the administrative cohorts (paediatric-onset MS versus matched cohort), only significant differences (at *P* < 0.05) reported

Year before index	ICD chapter	Chapter description	Events (PoMS cohort)	Events (matched cohort)	Rate ratio (95% CI)
1	1	Infection-related	12	19	3.10 (1.11–8.64)
1	2	Neoplasms	17	20	4.17 (1.50–11.61)
1	3	Endocrine and metabolic-related	32	41	3.83 (1.49–9.86)
1	6	Nervous system	51	28	8.94 (4.20–19.02)
1	7	Sense organs	143	39	18.00 (10.31–31.42)
1	17	Ill-defined signs and symptoms	169	96	8.64 (5.78–12.92)
1	19	Other health system contact	166	108	7.54 (5.59–10.18)
1	None	ICD code unassigned	39	87	2.20 (1.08–4.48)
2	19	Other health system contact	38	91	2.05 (1.00–4.19)
6	11	Genitourinary systems	9	9	5.01 (1.52–16.50)
7	2	Neoplasms	5	6	4.22 (1.03–17.29)
7	13	Skin-related	10	10	5.06 (1.91–13.40)
7	17	Ill-defined signs and symptoms	15	35	2.17 (1.01–4.64)
9	13	Skin-related	6	6	5.19 (1.18–22.88)
9	None	ICD code unassigned	45	56	4.17 (1.53–11.38)
10	19	Other health system contact	18	46	2.04 (1.05–4.00)
10	None	ICD code unassigned	36	76	2.47 (1.04–5.89)
16	1	Infection-related	7	9	4.33 (1.54–12.18)

#### Prescription drug classes dispensed

Similar to the ICD codes, not all therapeutic drug classes could be examined on a yearly basis (see Supplementary Table 2), but there was evidence of higher rates of prescriptions filled for the PoMS cohort up to 11 years before the index (Table 5). In the year pre-index, eight therapeutic drug classes were identified as being dispensed more frequently in the PoMS cohort: drugs for acid-related disorders, diabetes, vitamins, emollients and protectives, corticosteroids for dermatological use, systemic corticosteroids, antibacterials and anti-inflammatory and anti-rheumatic products (RRs ranged from 1.56 to 6.49). Four years prior, the RR for sex hormones and modulators of the genital system were higher (3.24, 95% CI: 1.36–7.71). There were no differences between groups from 5 to 9 years before the index, but 10 years before, dispensations for corticosteroids for dermatological use were higher (3.91, 95% CI: 1.54–9.91) and 11 years before, dispensations for cough and cold preparations were higher (2.48, 95% CI: 1.22–5.03). The number of events in the PoMS cohort contributing to each yearly RR ranged from 8 for diabetes and vitamins to 53 for systemic antibacterials.

**Table 5:** Annual rate ratios of prescription drug classes dispensed (at ATC 2nd level) in the administrative cohort (paediatric-onset MS versus matched cohort), only significant differences (at *P* < 0.05) reported

Year before index	ATC code	Prescription drug class by Anatomical Therapeutic Chemical (ATC) 2nd level	Events (PoMS cohort)	Events (matched cohort)	Rate ratio (95% CI)
1	A02	Acid-related disorders	17	22	3.76 (1.31–10.79)
1	A10	Diabetes	8	6	6.49 (1.39–30.25)
1	A11	Vitamins	8	7	5.56 (1.52–20.30)
1	D02	Emollients and protectives	11	20	2.68 (1.19–6.04)
1	D07	Corticosteroids (dermatological)	15	28	2.61 (1.30–5.24)
1	H02	Corticosteroids (systemic)	16	13	5.99 (2.87–12.49)
1	J01	Antibacterials (systemic)	53	165	1.56 (1.07–2.28)
1	M01	Anti-inflammatory and rheumatic	16	32	2.43 (1.32–4.48)
4	G03	Sex hormones and modulators of the genital system	12	18	3.24 (1.36–7.71)
10	D07	Corticosteroids (dermatological)	9	12	3.91 (1.54–9.91)
11	R05	Cough and cold preparations	11	23	2.48 (1.22–5.03)

## Discussion

In this population-based study of over 200 individuals with PoMS, we observed elevated patterns of healthcare use before the index date, i.e. before the clinically determined MS symptom onset date or first CNS demyelinating event. These elevations predominantly occurred 1–10 years pre-index, likely reflecting the prodromal period. In the administrative and clinical cohorts, healthcare encounters were higher for outpatient physician visits related to neoplasms, nervous system, sense organs, ill-defined signs and symptoms and ‘other health system contact’ in the year pre-index and for endocrine and metabolic disorder hospitalizations in the 5-year pre-index period. Between 2 and 10 years pre-index, there were measurable elevations in outpatient visits for which the physician could not, or did not, assign an ICD code, a pattern which was consistent in both cohorts. Prescription medication dispensations were also higher among PoMS than the matched individuals in both cohorts. These included corticosteroids (systemic and dermatological), measurable 1 and 10 years before the first CNS demyelinating event and 6 and 9 years before MS symptom onset. This study underscores the potential for early healthcare indicators of PoMS in the years preceding MS onset. Recognizing these patterns of elevated healthcare encounters could facilitate earlier detection strategies, providing an opportunity for timely interventions in at-risk individuals and potentially improving outcomes for those with PoMS.

The administrative and clinical cohorts shared a mean age at the index of 16 years. Many of the features of elevations in healthcare use pre-index dates were mirrored between the cohorts, although others differed. The potential implications of the patterns of healthcare use identified prior to each index date could also differ. Those identified before the first clinical recognition of a CNS demyelinating disease could be important in enabling earlier identification and potential intervention before an MS diagnosis. Visits related to the nervous system and sense organs were significantly elevated in the year immediately before the first CNS demyelinating code, possibly representing a missed opportunity for earlier diagnosis. There were also notable differences, predominantly between 6 and 10 years before this administrative index date, which included increased visits for skin-related issues and the genitourinary system. These findings align with existing literature in the general MS population, where dermatological and genitourinary visits are also elevated before the first medical recognition of MS or CNS demyelinating disorder, suggesting that these early healthcare encounters may serve as markers of prodromal MS.^13,14^

It is through the inclusion of the clinical cohort, for whom we had the date of MS symptom onset, that we may better understand the ‘true’ MS prodrome. In this cohort, visits related to neoplasms were elevated each year for 6 years before MS symptom onset, reaching statistical significance in years 1 and 4. While neoplasms are rare in children and adolescents^15^ (and the number of events was naturally small), findings could reflect underlying alterations in immunity.^16^ Findings could also reflect incidental identification, with a potential neoplasm discovered as part of a medical examination prompted by the presenting prodrome-related issues. Visits for which no ICD code was assigned, as well as injury-related and infection-related visits, were increased from 8 to 16 years before MS symptom onset. There is currently no established means of categorizing periods or events as prodromal or aetiological, but it is feasible that these events could indicate aetiological factors. Both trauma^17^ and infections^18^ have been identified previously as risk factors for the development of MS as well as potential markers of the MS prodrome.^13^ It is more challenging to interpret the visits with no assigned ICD code, but it seems likely that these represent symptoms, which were not readily identifiable in the ICD coding system. Along with findings that outpatient visits for ‘ill-defined signs and symptoms’ were elevated immediately prior to MS onset and recognition, suggests that these may represent ‘prodromal’ factors.

Paediatric-onset MS represents a small proportion of all MS cases, but the study of this cohort may offer the best opportunity to identify early signs of disease. Their younger age means that these individuals have likely had less exposure to negative health behaviours and accumulated fewer comorbidities compared with adults. To date, three studies were found which explored pre-disease healthcare use in the PoMS population.^6,19,20^ The first, a population-based study from Ontario, Canada, looked only at the year before the first CNS demyelinating disease claim and reported increased hospitalizations and physician visits among the PoMS cohort versus a matched non-MS cohort.^19^ However, the reasons (or diagnoses) behind these increased visits were not examined. In a tertiary health centre in St. Louis, USA, researchers reviewed the medical records of 21 PoMS patients before their first demyelinating claim. In the year before the index, they reported increased healthcare utilization (compared to the year prior within patients), particularly for neurology-related reasons, followed by infections, musculoskeletal and dermatological-related issues.^20^ Last, in a nationwide German study of >1000 PoMS patients identified using administrative data, the researchers identified distinct patterns of primary care use in the 5 years before MS recognition. Metabolic, ocular, musculoskeletal, gastrointestinal, and cardiovascular disorders were over-represented in the PoMS population (versus controls) during this period.^6^ Three of those 5 disorders with the greatest magnitude of difference between cases/controls were found in the ICD chapters for ill-defined signs and symptoms, one chapter that was consistently elevated before the index in the present study.

### Strengths and limitations

This was a nationwide study, including both an administratively and clinically identified cohort of individuals with PoMS. This allowed us to explore two definitions of paediatric-onset MS, each with their own strengths and limitations. While the date of MS symptom onset enables us to get closer to the ‘true’ MS prodrome, it could be subject to recall bias as it is recorded retrospectively by the neurologist based on a patient's medical history. In contrast, the health administrative derived index date is captured prospectively as the patient moves through the health system, though this information is not collected for research purposes and misclassification is possible. At this time, only administrative cohorts have been reported on in the PoMS population, so the inclusion of this cohort enabled comparisons with the literature.

An additional strength is that the prospectively collected longitudinal data allowed for the exploration of healthcare use up to 17 years before the index dates. Paediatric-onset MS, however, is relatively rare, leading to a modest sample size. This, combined with the cohorts’ young age, naturally led to few healthcare encounters for some conditions. This prevented analyses of some drug classes and ICD chapters, such as cardiovascular-related issues and limited ability to assess all years pre-index for all healthcare encounters. At the same time, extensive comparisons were performed, including by ICD chapter and by year, thereby increasing the potential for Type 1 error. Therefore, this work should be interpreted as being exploratory, with a need for further study to confirm or refute findings.

Further, we lacked data on primary care physician visits and signs or symptoms for which patients and their families did not seek healthcare, rates of which may have differed between groups. For those 28 PoMS patients identified in the administrative cohort and not the clinical registry, there is a small risk of misclassification as we cannot know whether they were formally diagnosed with MS (though they all had ≥3 visits to healthcare for MS, which carries a PPV of 98.8% in the wider MS population in Sweden,^12^ and 100% in the PoMS population in Ontario, Canada).^21^

While we had information on parental income as well as immigration status, we were not able to adjust for these factors in our models due to the low numbers of yearly events in each ICD chapter/prescription drug class. A study from the United States recently identified low neighbourhood-level socioeconomic status as a risk factor for PoMS,^22^ and immigration status can influence healthcare access, even in a universal healthcare setting.^23^ Our inability to adjust for these factors could have introduced confounding into our models. However, given the higher proportion of lower income families and immigrants in the PoMS versus matched cohorts (who typically use health services less), we believe that not adjusting for these factors likely biases our results towards the null. Meaning that our current results likely represent a conservative estimate of the true association.^23,24^

## Conclusions

In summary, we found that before MS onset and recognition, children and adolescents with MS exhibited elevated patterns of healthcare use and prescriptions dispensed that differentiated them from persons without MS. These differences primarily occurred 1 to 10 years before the clinically determined MS symptom onset or the first CNS demyelinating event identified in administrative data. Findings are suggestive of a prodromal period. These early indicators may offer insights into the pathophysiology of MS and aid in the early detection of PoMS.

## Supplementary Material

fcaf180_Supplementary_Data

## Data Availability

Requests for sharing of de-identified data will be considered on reasonable request and in accordance with current legislation regarding protection of personal data. The codes generated for the analyses are available in the Supplementary Materials.
