# Supplementary material for: Higher healthcare use before paediatric multiple sclerosis onset: a nationwide cohort study
Source: Brain Commun. 2025 May 7;7(3):fcaf180. doi: 10.1093/braincomms/fcaf180 (PMC12202005; doi:10.1093/braincomms/fcaf180)
Supplement: fcaf180_Supplementary_Data [file fcaf180_supplementary_data.docx]

**Supplementary Material**

| **No** | **ICD Chapters** | **Abbreviation** | **Diagnostic Codes** |
| --- | --- | --- | --- |
| 1 | Certain infectious and parasitic diseases | Infection-related | ICD-10: A00 – B99 |
| 2 | Neoplasms | Neoplasms | ICD-10: C00 – D49 |
| 3 | Endocrine, Nutritional And Metabolic Diseases, And Immunity Disorders | Endocrine & Metabolic-related | ICD-10: E00 – E89 |
| 4 | Diseases Of The Blood And Blood-Forming Organs | Blood-related | ICD-10: D50 – D89 |
| 5 | Mental, Behavioral and Neurodevelopmental disorders | Mental disorders | ICD-10: F01 – F99 |
| 6 | Diseases of the nervous system | Nervous system | ICD-10: G00 – G99 |
| 7 | Diseases of the sense organs | Sense organs | ICD-10: H00-H95 |
| 8 | Diseases of the circulatory system | Circulatory system | ICD-10: I00 – I99 |
| 9 | Diseases of the respiratory system | Respiratory systems | ICD-10: J00 – J99 |
| 10 | Diseases of the digestive system | Digestive system | ICD-10: K00 – K95 |
| 11 | Diseases of the genitourinary system | Genitourinary system | ICD-10:N00 – N99 |
| 12 | Pregnancy, childbirth and the puerperium | Pregnancy, childbirth | ICD-10: O00–O99 |
| 13 | Diseases of the skin and subcutaneous tissues | Skin-related | ICD-10: L00 – L99 |
| 14 | Diseases of the musculoskeletal system and connective tissues | Musculoskeletal system | ICD-10: M00 – M99 |
| 15 | Congenital anomalies | Congenital anomalies | ICD-10: Q00–Q99 |
| 16 | Certain conditions originating in the perinatal period | Perinatal-related | ICD-10: P00–P96 |
| 17 | Symptoms, Signs, And Ill-Defined Conditions | Ill-defined signs, symptoms | ICD-10: R00 – R99 |
| 18 | Injury and poisoning  Complication of surgical and medical care | Injury-related | ICD-10: S00 – T98  ICD-10: V00-Y99 |
| 19 | Factors Influencing Health Status and Contact with Health Services | Other health system contact | ICD-10: Z00 – Z99 |
| None | Missing or unassigned ICD Code | ICD code unassigned | None listed |

**Supplementary Table 1.** International Classification of Disease (ICD) Chapters and years for which they were analyzed in the administrative and clinical cohorts.

| **ATC 1st Level** | **ATC 2^nd^ Level** | **ATC 2^nd^ Level Description** |
| --- | --- | --- |
| **A**: Alimentary Tract and Metabolism | A01 | Stomatological Preparations |
|  | A02 | Drugs for acid-related disorders |
|  | A06 | Drugs for constipation |
|  | A10 | Drugs used in diabetes |
|  | A11 | Vitamins |
| **B**: Blood and Blood Forming Organs | B03 | Anti-anemic agents |
| **D**: Dermatologicals | D01 | Antifungals for dermatological use |
|  | D02 | Emollients and protectives |
|  | D06 | Antibiotics and chemotherapeutics for dermatological use |
|  | D07 | Corticosteroids, dermatological preparations |
|  | D10 | Anti-acne preparations |
| **G**: Genito-Urinary System and Sex Hormones | G02 | Other gynecologicals |
|  | G03 | Sex hormones and modulators of the genital system |
| **H**: Systemic Hormonal Preparations, Excluding Sex Hormones and Insulins | H02 | Corticosteroids for systemic use |
| **J**: Antiinfectives for Systemic Use | J01 | Antibacterials for systemic use |
|  | J05 | Antivirals for systemic use |
|  | J07 | Vaccines |
| **M**: Musculo-Skeletal System | M01 | Antiinflammatory and antirheumatic products |
| **N**: Nervous System | N02 | Analgesics |
|  | N03 | Antiepileptics |
|  | N05 | Psycholeptics |
|  | N06 | Psychoanaleptics |
| **R**: Respiratory System | R01 | Nasal preparations |
|  | R03 | Drugs for obstructive airway diseases |
|  | R05 | Cough and cold preparations |
|  | R06 | Antihistamines for systemic use |
| **S**: Sensory Organs | S01 | Ophthalmologicals |
|  | S03 | Ophthalmological and ontological preparations |

**Supplementary Table 2.** Anatomical Therapeutic Chemical (ATC) classification system 1^st^ and 2^nd^ levels. Only ATC 2^nd^ Level therapeutic classes which were analysed in at least one year are listed. Those with fewer than ten events amongst the PoMS and matched cohort combined or in which the statistical model did not converge, or the upper 95% CI went to infinity due to small number of events were not analysed/reported (i.e., no drug classes from the following ATC 1^st^ level groups were included: **C**: Cardiovascular System; **L**: Antineoplastic and Immunomodulating Agents; **P**: Antiparasitic Products, Insecticides, and Repellents; **V**: Various).

|  | *Clinical cohort* | | *Administrative cohort* | |
| --- | --- | --- | --- | --- |
|  | PoMS | Matched Cohort | PoMS | Matched Cohort |
| N | 140 | 686 | 141 | 686 |
| **Sex** |  |  |  |  |
| Female | 97 (69.3%) | 471 (68.7%) | 91 (64.5%) | 441 (64.3%) |
| Male | 43 (30.7%) | 215 (31.3%) | 50 (35.5%) | 245 (35.7%) |
| **Age at Index** |  |  |  |  |
| Mean (SD) | 16.0 (1.8) | 16.0 (1.8) | 15.9 (1.9) | 16.1 (1.9) |
| **Age at diagnosis** |  |  |  |  |
| Mean (SD) | 17.0 (2.3) | 17.1 (2.2) | 16.6 (2.1) | 16.8 (2.0) |
| **Age at Index (categories)** |  |  |  |  |
| <12 | 6 (4.3%) | 27 (3.9%) | 8 (5.7%) | 37 (5.4%) |
| 12-15 | 51 (36.4%) | 248 (36.2%) | 51 (36.2%) | 244 (35.6%) |
| 16-<18 | 83 (59.3%) | 411 (59.9%) | 82 (58.2%) | 405 (59.0%) |
| **Index Year (categories)** |  |  |  |  |
| 2001-2010 | 7 (5.0%) | 35 (5.1%) | 9 (6.4%) | 45 (6.6%) |
| 2011-2020 | 133 (95.0%) | 651 (94.9%) | 132 (93.6%) | 641 (93.4%) |
| **Socioeconomic status *** |  |  |  |  |
| 1(lowest income quintile, least affluent) | 17 (12.1%) | 36 (5.2%) | 14 (9.9%) | 47 (6.9%) |
| 2 | 20 (14.3%) | 64 (9.3%) | 20 (14.2%) | 49 (7.1%) |
| 3 | 23 (16.4%) | 99 (14.4%) | 25 (17.7%) | 92 (13.4%) |
| 4 | 31 (22.1%) | 165 (24.1%) | 30 (21.3%) | 169 (24.6%) |
| 5 (highest income quintile, most affluent) | 49 (35.0%) | 322 (46.9%) | 52 (36.9%) | 329 (48.0%) |
| **Immigrant status** |  |  |  |  |
| Immigrant | 8 (5.7%) | 14 (2.0%) | 12 (8.5%) | 20 (2.9%) |
| Non-immigrant | 132 (94.3%) | 672 (98.0%) | 129 (91.5%) | 666 (97.1%) |
| **Follow-up before index, years** |  |  |  |  |
| Mean (SD) | 8.4 (2.3) | 8.4 (2.3) | 8.8 (2.5) | 8.9 (2.5) |

**Supplementary Table 3.** Clinical and demographic characteristics of the administrative and clinical paediatric-onset multiple sclerosis (MS) and matched cohorts for the analysis of prescriptions dispensed.

* The socioeconomic status of the highest-earning parent was used. Eleven controls in the administrative cohort and 14 controls in the clinical cohort with missing parental socioeconomic status were assigned to category 3.SD: standard deviation, PoMS: pediatric-onset multiple sclerosis

**Code generated**

**R code to generate final models:**

summary(glm(Visits ~ Case_Ctrl+offset(log(Followup)), family="quasipoisson", data=DATA))


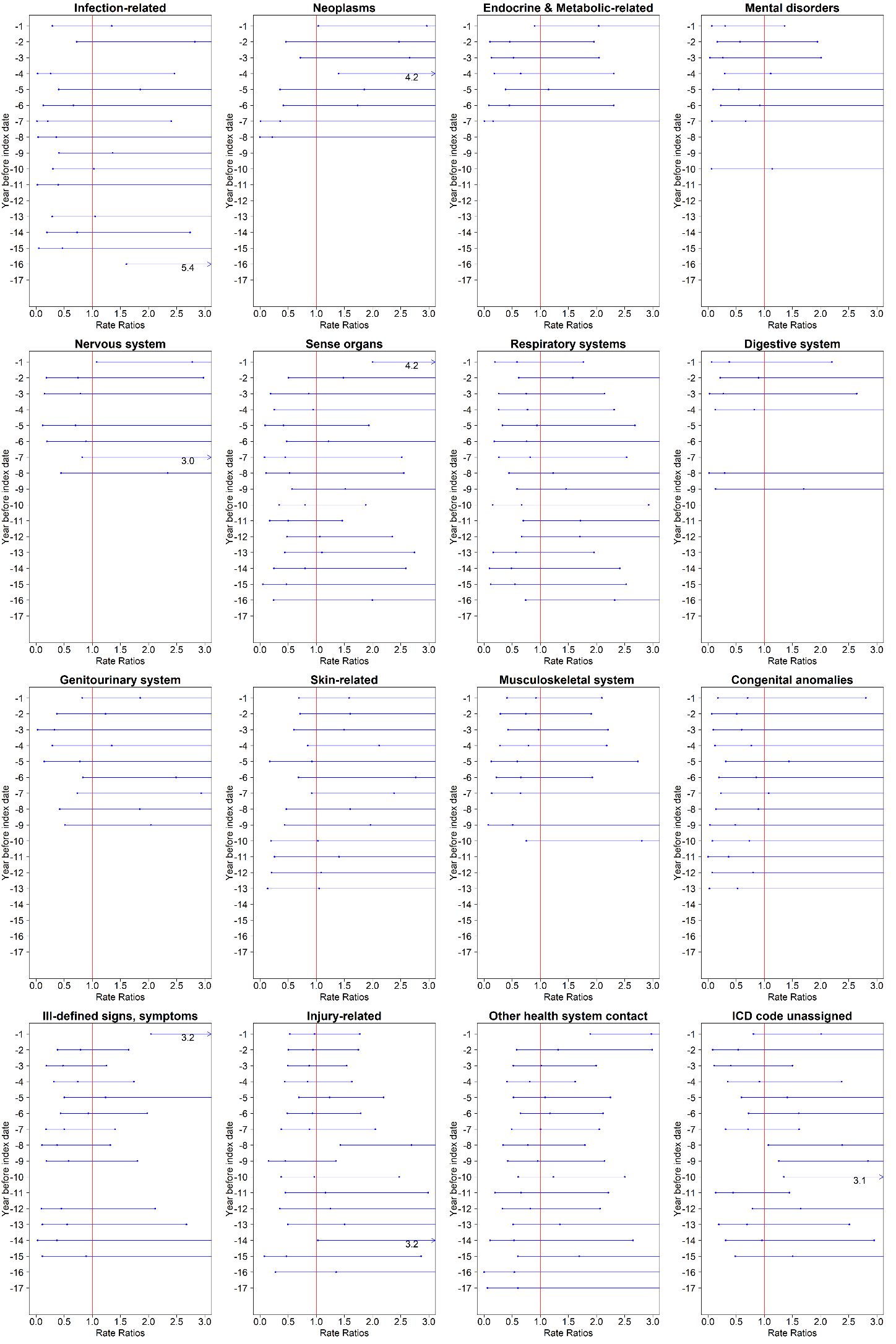


**Supplementary Figure 1. Rate ratios (RRs) and 95% confidence intervals of outpatient visits by ICD chapter amongst the clinical cohort.** The Y-axis represents the years from the index date from the first year pre-index (-1) to 17 years pre-index (-17). The X-axis represents the risk ratio (RR). RR >1 indicates a higher number of outpatient visits among the PoMS versus the matched cohort. The arrow indicates an RR > 3. Chapters with too few events (<10 events for the MS and matched cohort combined) or in which the model did not converge in any year were not analysed/depicted here. These included ICD chapters for the circulatory system, pregnancy, childbirth, and perinatal-related.


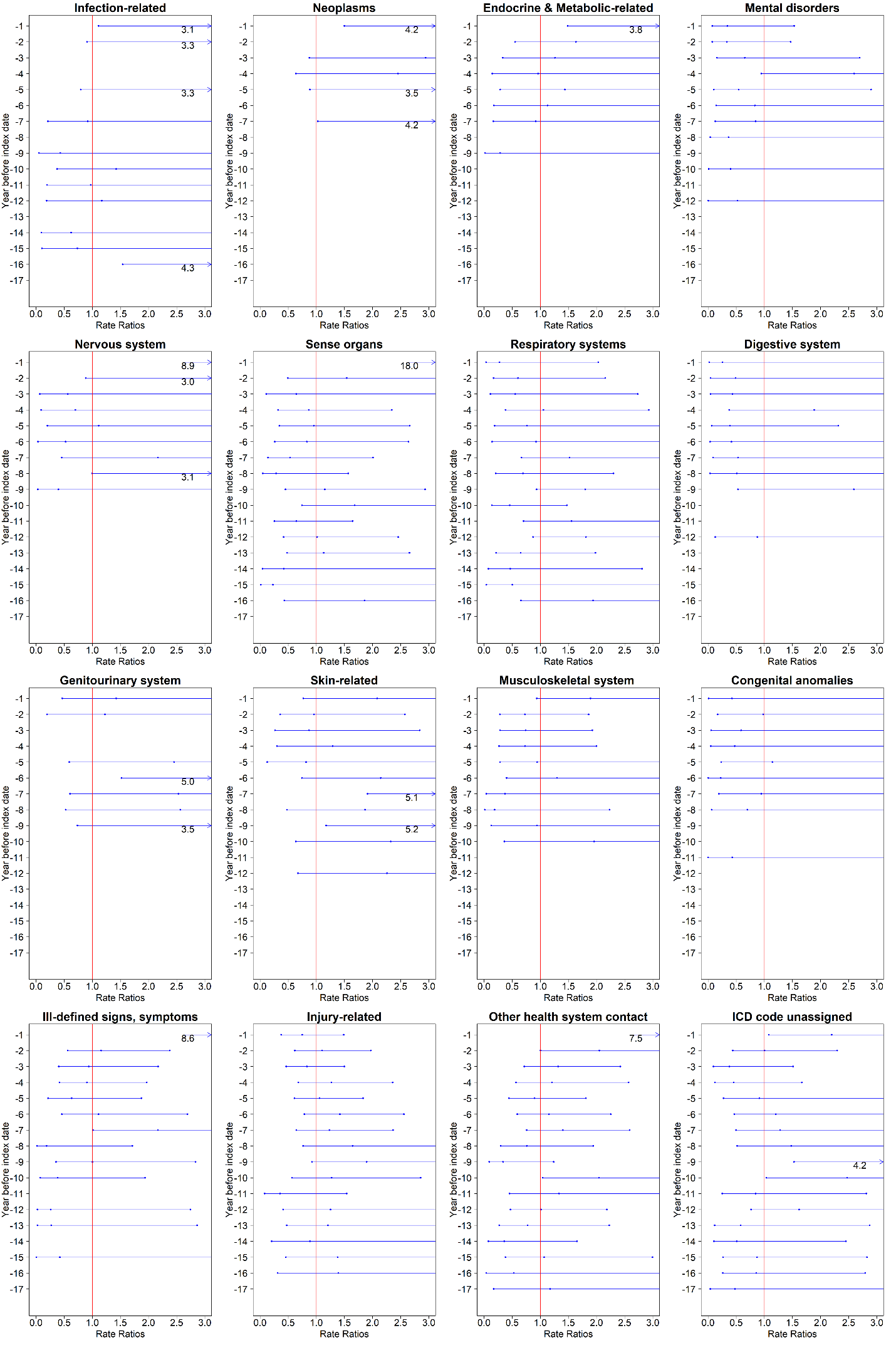


**Supplementary Figure 2. Rate ratios (RRs) and 95% confidence intervals of outpatient visits by ICD chapter amongst the administrative cohort.** The Y-axis represents the years from the index date from the first year pre-index (-1) to 17 years pre-index (-17). The X-axis represents the risk ratio (RR). RR >1 indicates a higher number of outpatient visits among the PoMS versus the matched cohort. The arrow indicates an RR > 3. Chapters with too few events (<10 events for the MS and matched cohort combined) or in which the model did not converge in any year were not analysed/depicted here. These included ICD chapters for the circulatory system, pregnancy, childbirth, and perinatal-related.
